# The Interscutularis Muscle Connectome

**DOI:** 10.1371/journal.pbio.1000032

**Published:** 2009-02-10

**Authors:** Ju Lu, Juan Carlos Tapia, Olivia L White, Jeff W Lichtman

**Affiliations:** 1 Department of Molecular and Cellular Biology, Harvard University, Cambridge, Massachusetts, United States of America; 2 Center for Brain Science, Harvard University, Cambridge, Massachusetts, United States of America; 3 Department of Physics, Massachusetts Institute of Technology (MIT), Cambridge, Massachusetts, United States of America; The Rockefeller University, United States of America

## Abstract

The complete connectional map (connectome) of a neural circuit is essential for understanding its structure and function. Such maps have only been obtained in *Caenorhabditis elegans.* As an attempt at solving mammalian circuits, we reconstructed the connectomes of six interscutularis muscles from adult transgenic mice expressing fluorescent proteins in all motor axons. The reconstruction revealed several organizational principles of the neuromuscular circuit. First, the connectomes demonstrate the anatomical basis of the graded tensions in the size principle. Second, they reveal a robust quantitative relationship between axonal caliber, length, and synapse number. Third, they permit a direct comparison of the same neuron on the left and right sides of the same vertebrate animal, and reveal significant structural variations among such neurons, which contrast with the stereotypy of identified neurons in invertebrates. Finally, the wiring length of axons is often longer than necessary, contrary to the widely held view that neural wiring length should be minimized. These results show that mammalian muscle function is implemented with a variety of wiring diagrams that share certain global features but differ substantially in anatomical form. This variability may arise from the dominant role of synaptic competition in establishing the final circuit.

## Author Summary

Conventionally, the organization of a neural circuit is studied by sparsely labeling its constituent neurons and pooling data from multiple samples. If significant variation exists among circuits, this approach may not answer how each neuron integrates into the circuit's functional organization. An alternative is to solve the complete wiring diagram (connectome) of each instantiation of the circuit, which would enable the identification and characterization of each neuron and its relationship with all others. We obtained six connectomes from the same muscle in adult transgenic mice expressing fluorescent protein in motor axons. Certain quantitative features were found to be common to each connectome, but the branching structure of each axon was unique, including the left and right copies of the same neuron in the same animal. We also found that axonal arbor length is often not minimized, contrary to expectation. Thus mammalian muscle function is implemented with a variety of wiring diagrams that share certain global features but differ substantially in anatomical form, even within a common genetic background.

## Abstract 3

Reconstruction of the complete set of motor axons innervating a muscle reveals wiring variability, even among corresponding neurons on the left and right sides of the same animal.

## Introduction

The nervous system's connectivity is believed to be a fundamental determinant of its function [1,2], but in general it is not readily accessible. One way to characterize neural circuits is to extract statistical properties of connectivity, often by pooling data from multiple animals [3–6]. This method assumes that connectional specificity at the level of classes of cells suffices to account for the properties of circuits [7–9]. It also assumes that within a class, each neuron's connectivity is established independently, without correlations with that of other cells. While such models may provide interesting ideas about how the nervous system works, their underlying assumptions are probably oversimplified. Neurons, for example, often innervate a nonrandom subset of cells within their target, rather than stochastically innervating such a group of cells [10,11]. In many circuits, neurons innervating the same group of cells do not establish connections independently, as evidenced by interneuronal competition observed during development [12,13]. Thus some neuroscientists have concluded that “any attempt to interpret neuronal connectivity purely in terms of probabilities … must be doomed to failure [14].” The obvious alternative is to obtain complete wiring diagrams (connectomes) of either the entire nervous system of an individual animal, or a well-defined subnetwork of the nervous system, based on direct observation rather than statistical inference. It is possible that such maps might ultimately reveal that neural circuits are stochastic in certain aspects and thus amenable to probabilistic descriptions. On the other hand, such maps may reveal organizational specificity that may not be detectable by statistical analysis, especially when the structure and connectivity of individual neurons need to be characterized in the context of the entire circuit (see below).

The first attempt to directly describe a connectome was undertaken in the parasitic nematode Ascaris lumbricoides with optical microscopy [15–17], but it produced only “enigmatic wiring diagrams” [18] because of inadequate resolution. The only successful connectomic reconstruction was accomplished in another nematode, C. elegans, using serial electron microscopy [2,18–20]. This map has proven to be a valuable resource for further analysis of circuits underlying C. elegans behaviors [21–23]. Therefore, it is likely that mammalian connectomes will also provide important information.

The advent of transgenic technologies to label neurons [24], combined with automated optical microscopy and computer-assisted image analysis tools [25], provides an avenue for the reconstruction of mammalian connectomes. Nevertheless, given the enormous complexity of mammalian nervous systems, it is necessary to begin this endeavor with tractable circuits. In this work we attempt to generate the complete wiring diagram of a peripheral neuromuscular circuit. This circuit consists of the full set of α-motor axons and the full complement of muscle fibers in the single muscle innervated by these axons. It can be captured in its entirety because each muscle's innervation is nonoverlapping. In contrast, any finite volume of circuitry in the central nervous system (CNS) contains neuronal processes entering and leaving the volume, so completeness of reconstruction cannot be achieved locally. Another advantage of the neuromuscular circuit is that its functional organization has been studied intensively, which culminated in the discovery of the size principle [26], namely, the recruitment of motor neurons proceeds in the order of increasing twitch tensions. The anatomical underpinnings of the graded tensions elicited by the group of motor neurons, however, have not been demonstrated. An additional rationale for studying the neuromuscular connectome is that the mature wiring diagram emerges from an extensive postnatal reorganization of axonal arbors known as synapse elimination. Previous imaging studies [27,28] suggested that the fate of different axons that co-innervate the same NMJ is influenced by the interactions of these axons at other NMJs with other axons. Therefore, predicting which branches are retained and which are pruned requires analyzing the competitive relationships among the entire group of neurons. In this work we took the first step of unraveling the rules of this competition by generating the adult neuromuscular connectome, which is the end product of this aforementioned process. Lastly, comparing corresponding connectomes between different animals or in the same animal (e.g., left versus right side) may help clarify the extent to which genetics, epigenetic factors, and random fluctuations impact circuit structure.

## Results

**Figure 1:**
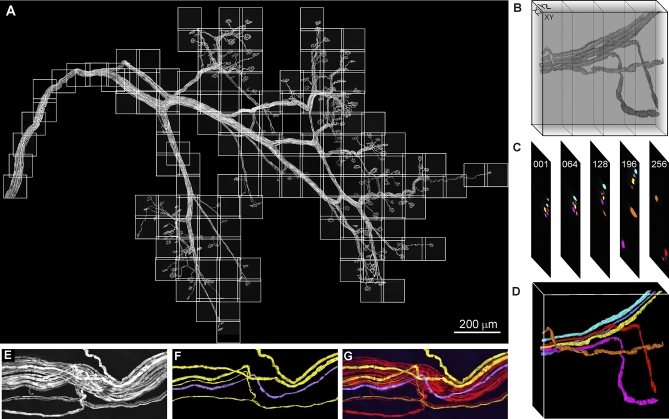
Method for Reconstructing Neuromuscular Connectomes (A) All the axons (labeled with YFP) in an interscutularis muscle were imaged by confocal microscopy with motorized stage. A montage of 146 overlapping image stacks (white squares) provided the dataset (total ∼43 GB) for connectome reconstruction. (B–D) Each XY stack (B) was digitally resampled along an orthogonal axis (*x* or *y*) so that most axons could be followed in cross-sections (C). Axonal contours in cross-sections were delineated, color-coded, and rendered in 3D (D) using semi-automatic tracing software. (E–G) Reconstruction accuracy was tested by tracing motor axons in a muscle from a triple transgenic mouse in which all axons expressed an orange fluorescent protein (KOFP) and a small subset of axons expressed in addition either CFP or YFP. (E) Appearance of the nerve in the KOFP channel. From an adjacent section we selected several doubly labeled axons and reconstructed them in this volume using the KOFP channel only. (F) Monochromatic reconstruction results for doubly labeled axons. Lavender, KOFP + CFP; yellow, KOFP + YFP. (G) All three fluorescent channels shown for the volume. The results were identical for the doubly labeled axons (compare [F] and [G]).

**Figure 2:**
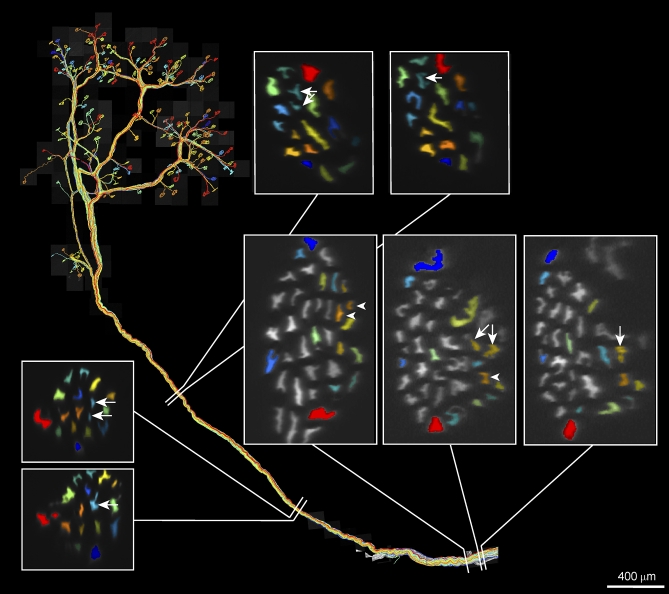
Extramuscular Branching of Motor Axons Projecting to the Interscutularis Muscle All axons innervating sample M4R were reconstructed and color-coded according to their motor unit sizes (see Figure 3). Axons shown in grey projected to other muscles and did not innervate the interscutularis muscle. White boxes show the nerve's cross section just before and just after each of the four instances of axonal bifurcation (arrows and arrowheads show the branching axon).

**Figure 3:**
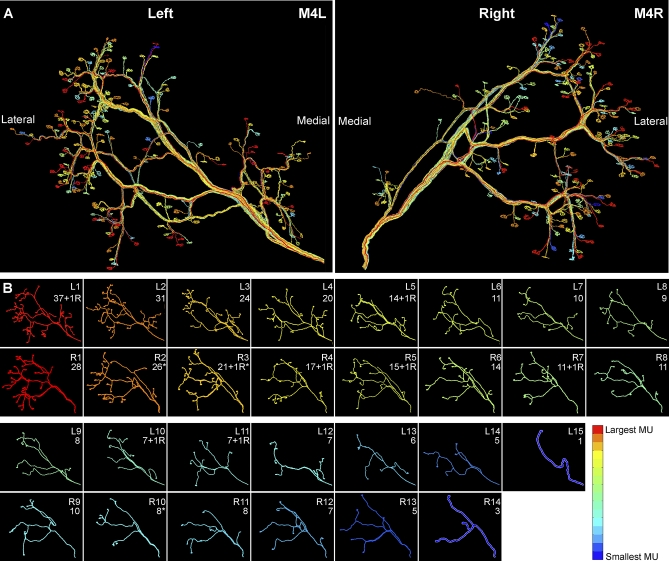
Connectomes of a Left-Right Pair of Interscutularis Muscles (A) Axons in the connectomes of the left (L) and right (R) interscutularis muscles of a 1-mo-old animal (M4) were color-coded based on the rank-order of their motor unit sizes in each connectome. (B) Comparison of the branching pattern of each axon with its contralateral counterpart from the largest (L1 and R1) to the smallest (L15, R14). Axons in M4R were flipped horizontally to facilitate visual comparison. Lower numbers in each panel indicate motor unit sizes. R, retraction bulb; *, one NMJ co-innervated by two axons.

We chose to study the connectome of the mouse interscutularis, a muscle that attaches to the base of the ear and to the middle of the skull, because it is small, very thin, and is innervated by relatively few neurons. We used YFP-16, one of the few transgenic mouse lines that express fluorescent protein in 100% of motor neurons [24] to catalogue every motor unit in individual muscles (recently developed *Brainbow* lines do not label all motor neurons; see [29]). With confocal microscopy and semi-automated 3D reconstruction tools (Figure 1), we obtained complete connectomes of six interscutularis muscles in four mice (e.g., Figures 2 and 3, and Figure S1). Typical datasets for one muscle consisted of ∼150 image stacks, each stack containing on average ∼150 images (16 bit, 1K × 1K), totaling ∼40 GB of data. The accuracy of this reconstruction method was confirmed in three different ways (Figure 1E–1G and see Materials and Methods).

Because motor axons may branch en route to the target muscles [30], in some samples we followed each axon's trajectory far back (up to ∼0.5 cm) along the posterior auricular branch of the facial nerve (cranial nerve [CN] VII). We found that 6% (10/162) of these traced axons branched extramuscularly (Figure 2). Among these ten axons, three branched <1 mm away from where they entered the muscle, three branched 1–2 mm away, two branched 2–3 mm away, and two branched >3 mm away. None of these axons branched more than once, and all branches eventually entered the interscutularis muscle. Furthermore, we found no tendency for the axons to branch at any particular sites, such as sites where the nerve branched to supply other muscles. These data support the idea that extramuscular branching in each axon occurred independently.

### The Interscutularis Connectome

The reconstructed interscutularis connectomes (Figure 3 and Figure S1) provided an atlas of neuromuscular connectional diagrams of all the axons within the muscle. This atlas included information about the number and position of all the postsynaptic targets, as well as branching topology, neighbor relations, and segmental geometry of each axon. Some of the information, such as motor unit sizes (number of neuromuscular junctions [NMJs] innervated by one axon) and statistical properties of axonal tree structures, may be obtainable by pooling single axon data from many sparsely labeled muscle samples, if homogeneity among animals and unbiased sampling are assumed. In this case, the connectomic approach provides a compendium of such information efficiently. More importantly, however, other aspects of neuromuscular circuit organization, such as neighbor relations in the fasciculation and innervation pattern, can only be understood by placing each individual axon into the context of the whole circuit's structure, and thus require the connectomic approach. Moreover, comparison between identified neurons across mice cannot be achieved by random, sparse labeling (see below).

**Figure 4:**
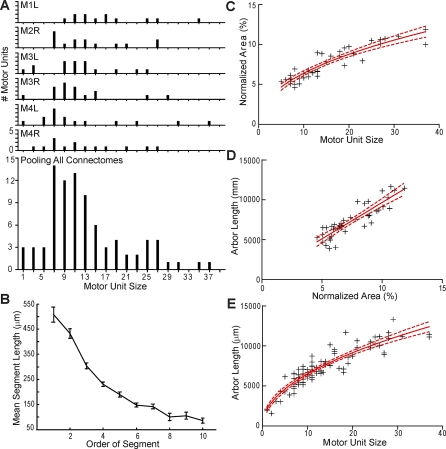
Common Features Exhibited by All Connectomes (A) Motor unit size distribution in each muscle was skewed towards smaller motor units, in agreement with previous physiological data (see text). (B) Mean axonal segment length decreased as segment order increased (71 axons from five muscles, see Figure S3 for more details). Error bars, standard error of the mean (SEM). (C) Normalized axonal cross-sectional area *A* scaled as the square root of motor unit size *M* (*A* ∼ *M* ^0.4544^, 95% CI of slope: 0.3903–0.5185, *R* ^2^ = 0.8445; *n* = 40). In (C–E) red solid lines are best fitting; red dotted lines show 95% CI. (D) Axonal arbor length *L* scaled linearly with *A* (*R* ^2^ = 0.7775; *n* = 40). (E) *L* scaled as the square root of *M* (*L* ∼ *M* ^0.4938^, 95% CI of slope: 0.4428–0.5447, *R* ^2^ = 0.8394; *n* = 87).

To summarize the data: each connectome contained 14.5 ± 1.5 axons and 198 ± 11 muscle fibers. The axons exhibited a wide range of motor unit sizes, with a predominance of smaller motor units over larger ones (Figure 4A). The smallest motor units had only one NMJ (2/87 axons); the largest motor units had 37 NMJs (2/87 axons).

We found that among the 979 instances of axonal branching, most (88.5%) were binary, with progressively smaller fractions of higher degree branching (tri-furcations 10.7%, 4-furcations 0.6%, 5-furcations 0.2%, see Figure S2). We then analyzed branching symmetry of axons that innervated more than three NMJs (*n* = 83). This symmetry was evaluated with the imbalance index *I* [31], which is 0 for a completely symmetric tree (e.g., each branching point gives rise to exactly two daughter branches), and one for a completely asymmetric tree (e.g., each branching point gives rise to one terminal branch and one branch that further bifurcates). Most axons were relatively symmetric (*I* = 0.31 ± 0.21), and axons with larger motor unit sizes tended to be more symmetric (Spearman test, *p* < 0.0001).

The total intramuscular length of axonal arbors ranged from 1,583 μm to 13,320 μm (7,256 ± 2,352 μm, mean ± standard deviation [SD]), with a positive correlation with motor unit sizes. Axonal segments between branching nodes became progressively shorter with increasing branch orders (Figures 4B and S3).

We noted that the range of motor unit sizes (7.7 ± 2.8-fold) was similar to that of twitch tensions recorded in previous physiological studies of mammalian muscle contraction (e.g., 8.3-fold [32], 12.3-fold [33]). Furthermore, both the twitch tension distribution and the motor unit size distribution shared the same shape: unimodal and skewed towards the smaller end (Figure 4A). These results strongly argue that motor unit sizes are the anatomical underpinning of the observed distribution of twitch tensions. As the entire collection of motor units in each muscle was known in our dataset, we could ask whether all connectomes follow the same motor unit size distribution. Indeed we found that motor units in all six connectomes were distributed in the same way (*p* > 0.2, Kruskal-Wallis test). Given that motor neurons are recruited in a fixed order (weak to strong, see [26]), the correspondence between motor unit size and twitch tension mentioned above allowed us to establish the functional correspondence between individual axons in different muscle samples.

Based on conduction velocity studies, axons generating larger twitch tensions appear to possess larger calibers [33,34]. We thus anticipated that axonal cross-sectional area should correlate with motor unit size. We measured the mean cross-sectional area of each axon right before its first intramuscular branch and normalized the area to the total cross-sectional areas of all axons innervating the same muscle. We found that the normalized cross-sectional area *A* was correlated with the motor unit size *M* obeying a power law: *A* scaled approximately as the square root of *M* (Figure 4C). Furthermore, the cross-sectional area of first order axonal branches was correlated with the number (*N*) of downstream NMJs by a similar scaling relationship: *A* ∼ *N* ^0.536^, (*n* = 47, 95% confidence interval [CI] of the exponent: 0.4663–0.6051). This similarity suggests that the scaling relationship is a fundamental property of motor axon branching.

In order to better understand the origin of this relationship, we measured the total axonal arbor length *L* distal to the point where the axon enters the muscle. We found that *L* scaled linearly with *A* (Figure 4D). Furthermore, *L* also scaled as the square root of *M* (Figure 4E). Taken together, these results support the idea that the principal determinant of axonal cross-sectional area is the energy cost associated with axonal membrane (see Discussion for details).

As the arrangement of different motor units in a muscle affects the mechanical properties of force delivery, we proceeded to address how motor units are deployed relative to each other in the interscutularis. The positions of NMJs in most motor units were distributed uniformly in the endplate band (Figure S4), both across the width of the muscle (80/83, 96.4%) and along the muscle's length (79/83, 95.2%). Statistical test also suggested that some motor units (18/83 across the muscle, 4/83 along the muscle) were “super-uniform,” i.e., the distribution was too regular to be from a random uniform sample. Therefore, the interscutularis muscle does not seem to possess compartments as described in certain larger muscles [35,36].

In distinction to entire motor units, primary subtrees of individual axons were not uniformly distributed. In most cases they appeared to invade nonoverlapping territories (for example, see Figure S5). We compared the distribution of subtree terminals of 27 axons in which each subtree had at least four terminals. We found that in 20 axons (74%) the distribution of terminals of the two subtrees was different (*p* < 0.05, generalized Wald-Wolfowitz test [37]). In particular, in 12 axons (44.4%) the territories of the two subtrees were completely segregated. On the other hand, when primary subtrees belonging to different axons were compared, their territories tended to be overlapping (78/112 pairs, 69.4%, *p* < 0.05, generalized Wald-Wolfowitz test). This arrangement of subtrees suggests that developmental mechanisms prevent multiple branches of the same axon from projecting to the same region, while permitting branches of different axons to intermingle in the same region. Such mechanisms may explain the observation that multiple axons innervate the same muscle fiber at early developmental stages [13], whereas rarely do two branches of the same axon innervate a single muscle fiber.

### Nerve Fasciculation Patterns

**Figure 5:**
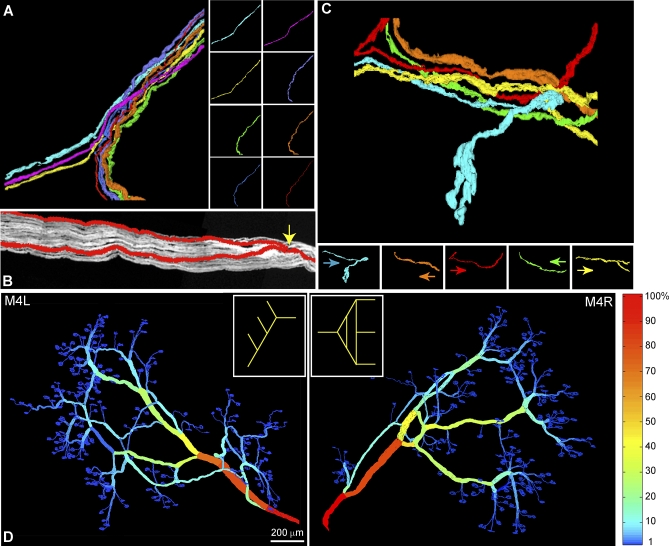
Nerve Fasciculation Pattern and Its Relationship to Axonal Branching (A) A nerve fascicle branched while its constituent axons did not branch. (B) An axon branched within a nerve fascicle. The two resultant branches continued in parallel in the same nerve. (C) Five axonal branches traveled in opposite directions in the same nerve fascicle. Arrows indicate the proximal-distal direction of each branch. (D) Nerve fasciculation patterns were not symmetrical in the left-right pair of muscles. Each nerve fascicle was color-coded according to its innervation weight, which was defined as the percentage of a muscle's NMJs innervated by axonal branches traveling through the fascicle. Insets: a skeleton of these two connectomes (M4L and M4R) were obtained by removing all fascicles with weights less than 10%. The topologies of the two skeletons were different. For example, there were no loops in M4L but two loops in M4R.

The intramuscular nerve fasciculation patterns reflect the collective behavior of all the axons. We found that the relationship between branching structures of individual axons and nerve fascicles was surprisingly complicated. Individual axons' branching behavior was not strictly coupled to the fasciculation pattern of the nerve. At some nerve branching points no axons branched; different axons simply followed one of the paths (Figure 5A). On the other hand, some axons branched inside a nerve segment and the resultant branches traveled in parallel along the same segment over some distance (Figure 5B). The most conspicuous example of such behavior was the extramuscular branching of axons discussed previously (Figure 2). Moreover, although most fasciculated nerve segments travel in a proximal-distal direction, some axonal branches contained in them did not follow the same direction. For example, in Figure 5C three axons entered the nerve fascicle from the left and two axons traveled in the opposite direction. Taken together, among 85 branching axons, 69 (81.2%) branched at least once within a nerve segment, and 29 (34.1%) contained at least one branch that traveled against the direction of some nerve segments. Overall, 89.4% of the axons deviated in some way from being a proper subgraph of the nerve fasciculation pattern.

It is possible that intramuscular nerve fasciculation reflected predetermined patterning similar to the highly stereotyped nerve structures seen more proximally (e.g., the brachial plexus). We thus tested whether there might be a conserved core fasciculation pattern in the interscutularis muscle. We assigned to each segment of the nerve a weight proportional to the total number of downstream NMJs (Figure 5D). We found that the extracted “skeletons” were topologically distinct in each muscle including left-right pairs in the same animal (Figure 5D insets). Therefore it seems unlikely that nerve fasciculation patterns in a muscle are genetically predetermined.

### Variability between Connectomes in Different Interscutularis Muscle Samples

As mentioned above, the knowledge of motor unit sizes of all axons allowed us to identify exact neuronal counterparts in different muscle samples. This knowledge enables exploration of a question that has been investigated in invertebrates but, to our knowledge, never in terrestrial vertebrates: the degree to which an individually identified neuron shares the same branching structure with its counterparts in other samples. Although nerve fasciculation patterns differed from sample to sample as shown above, the possibility that the branching structures of axonal counterparts be identical could not be ruled out, as axonal branching structures are not necessarily subgraphs of the nerve fasciculation pattern (see section above).

In order to compare neuronal counterparts, we first determined whether there were systematic differences between left and right copies of the interscutularis muscle. The number of muscle fibers on left and right sides was not significantly different (left 201.7 ± 20.7 versus right 199.0 ± 18.0, *n* = 6 pairs, two-tailed *p* = 0.53, paired Student's *t*-test; Figure S6A), nor was there a difference in the distribution of muscle fiber types (type I: left 40.3%, right 42.4%; type IIA: left 20.1%, right 19.1%; type IIB+IIX: left 39.6%, right 38.5%; two pairs). The number of innervating motor neurons was not significantly different either (left 14.7 ± 1.5 versus right 14.0 ± 1.4, six pairs, two-tailed *p* = 0.47, paired Student's *t*-test; Figure S6B). We thus proceeded to identify each neuron and its counterparts based on motor unit size and/or its rank within the connectome.

We analyzed four connectomes in two animals (left-right pair for each animal). We first compared the largest motor unit with its contralateral counterpart in the same animal. In one case their sizes were similar (animal M3, left 25 NMJs [12.8% of all NMJs in the muscle] versus right 29 [14.8%], Figure S1B), but in the other case less so (animal M4, left 37 [18.8%] versus right 28 [15.2%], Figure 3B). Moreover, their appearances did not exhibit appreciable similarity upon visual inspection (e.g., Figure 3B, L1/R1 pair). We then compared smaller motor units with their contralateral counterparts and again found no appreciable similarity in the branching structures. Whether the counterpart was defined by rank order (Figures 3B and S1B) or by absolute motor unit size made no difference; in each case there was no evidence for a common branching pattern.

**Figure 6:**
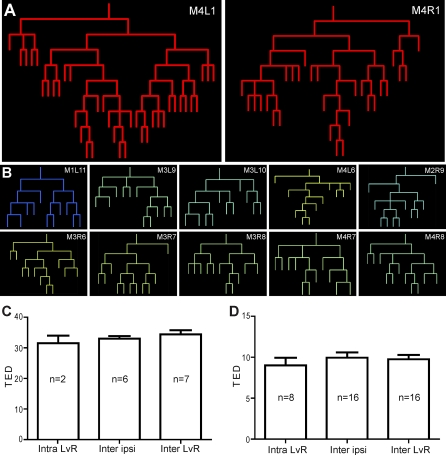
Topological Variability among Corresponding Axons (A) The largest motor units (M4L1 and M4R1), shown as dendrograms, exhibited different topological structures. (B) Ten motor units with identical motor unit size (11 NMJs) from six muscles all had different topologies. The color differences between panels indicate that these axons ranked slightly differently in their respective connectomes. (C) TED between the largest motor units in six muscles was not significantly different among the three groups: intra-animal left versus right pairs, inter-nimal ipsi-lateral (left-left or right-right) pairs, and interanimal left versus right pairs (one-way ANOVA, *p* = 0.45). (D) TED between medium-sized axons with the same motor unit size (*n* = 11 NMJ) in six muscles was not significantly different among the same three groups (one-way ANOVA, *p* = 0.67). Error bars: standard deviation.

In order to investigate whether left-right pairs of axons with the same rank or same motor unit size are similar in less obvious ways, we focused on their topologies, ignoring geometric features (e.g., length and angle of branches). We found a wide range of different topologies between axons with the same rank (Figure 6A) and even among axons with the same motor unit size (Figure 6B). We used tree-editing distance (TED [38]) to quantify the topological difference between axons. We found that left-right pairs of axons in the same animal (intra-animal pairs) were no more similar to each other than interanimal pairs of same-sized axons (Figure 6C). Furthermore, intra-animal pairs of axons were no more similar than pairs of synthetic “axons” randomly selected from an ensemble of tree structures generated by a Monte Carlo simulation (Figure 6D) (intra-animal pair TED, 9.00 ± 2.62; Monte Carlo TED, 8.87 ± 2.50; two-tailed *p* = 0.89; unpaired Student's *t*-test). These results indicate that the topologies of intra-animal left-right pairs of axons were not correlated.

### Suboptimality in Wiring Length

**Figure 7:**
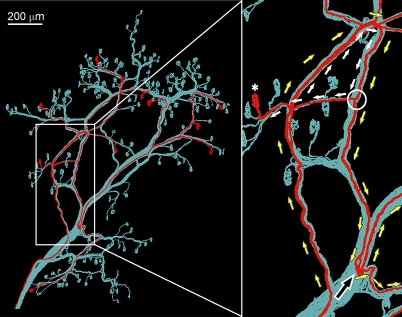
Suboptimality of Wiring Length of Individual Axons Wiring of a motor axon (red, M4L6, see Figure 3B) was superimposed on the nerve fascicles (blue) of the entire muscle. (Right) The axon took a long detour (yellow arrows) even though a much shorter path existed (black arrow). In addition it did not directly branch to innervate the NMJ to the left (*) but only reached it after looping back (white arrows) and bifurcating at the white circle. This kind of suboptimality in wiring length was observed in almost all connectomes.

In addition to the variability of branching topology we found that axonal trajectories did not adhere to the principle of minimization of total wiring length, initially proposed by Cajal [1] and supported by the full reconstruction of the C. elegans nervous system [39–41] but see [42]. Even superficial visual inspection of the interscutularis connectome showed that almost every axon's total length could be shortened by following different nerve fascicles or altering the location of branching points. Remarkably, some axons took highly tortuous routes to their target muscle fibers even when more direct paths seemed possible (Figure 7). Moreover, ∼6% of axons branched extramuscularly as previously mentioned (Figure 2), which is also suboptimal, as the two resultant branches of the same axon invariably continued together into the same muscle. In these cases, removal of the extramuscular branching could have saved 2,072 ± 1,116 μm (*n* = 4 axons) of wiring length, which is equivalent to 25 ± 9.5% of the intramuscular wiring length of these axons. As the intramuscular wiring length is comparable to the distance from the cell body to the muscle, its contribution to the total metabolic cost of the cell is substantial. Therefore, the 25% extra wiring length imposes significant additional metabolic load to the cell. However, from the perspective of the neuromuscular system as a whole, this additional cost may be insignificant, since the metabolic load of muscle contraction far exceeds that of axonal conduction.

## Discussion

There are several reasons for mapping the connectome, i.e., the entire wiring diagram of a neural circuit. Most importantly, this map represents the complete inventory of connectional information in one particular sample. The conventional approach to circuit analysis, in contrast, infers connectivity by pooling partial data from many samples, and thus relies on assumptions such as homogeneity of neurons in a population, or stereotypy of circuits among different individuals. The connectomic approach is advantageous because it does not require such assumptions. Moreover, as every cell is identified, the way in which each cell integrates into the organization of the circuit is revealed. Lastly, comparison between different instantiations of the same circuit can reveal those aspects of connectivity that are physiologically relevant, and those that are not.

In this work we present an initial attempt to reconstruct mammalian subnetwork connectomes. We chose the mouse interscutularis muscle as the starting point because of its simplicity and accessibility. Its simplicity lies in the fact that being an end organ, it does not have strong recurrent components. This feature allows us to achieve “completeness” within a finite volume, as opposed to the situation in most CNS circuits, where recurrent connections originating from distant sites are commonplace. Furthermore, it is simple because the input is purely divergent: each axon innervates multiple muscle fibers but each muscle fiber has only one input. This pattern is only present at a few places of the CNS.

Although the interscutularis muscle represents one of the smallest possible connectomes in mammals, it still presented significant technical challenges for reconstruction. Often axonal branches were tightly fasciculated with each other, the distance between which approached the resolution limit of confocal microscopy. This problem was aggravated by scattering especially when imaging deeper structures. Therefore axonal profiles sometimes bled into each other, so computer segmentation had to be monitored and complemented by manual intervention, which significantly reduced the speed of reconstruction. Optimally, the *Reconstruct* program we modified for automatic segmentation traced out 4 mm of axonal length per hour. In practice, however, the requirement of human monitoring and editing reduced it to ∼0.5 mm per hour.

We anticipate that the automated imaging and semi-automated reconstruction undertaken in this work will also be generalized to the study of CNS connectomes. However, the aforementioned technical difficulties in imaging and image analysis would be greater in the CNS, where the length scale of neural structures is much smaller and the packing of neuropil is much denser. Thus future technical innovations are required to facilitate fully automated reconstruction. For example, different colors may be introduced to spectrally separate different neurons [29]; imaging resolution may be improved through super-resolution techniques [43–46]; aberrations induced by scattering in deep tissues may be overcome by serial sectioning followed by either electron microscopy [47,48] or optical microscopy [49], by adaptive optics [50], or by tissue clearing [51].

The reconstructed connectomes demonstrated four organizational principles of neuromuscular circuits. First, the motor unit size distribution in each connectome paralleled previous results from physiological recordings of twitch tensions, providing an anatomical correlate for Henneman's size principle, which until now was a physiological concept. The skewed distribution of twitch tensions, obtained by pooling data from many different samples, demonstrated that statistically most motor units generate small twitch tensions, and a few generate large twitch tensions. However, the degree to which the set of motor units within each muscle obeys the same distribution has not been directly demonstrated. Our measurement of all motor units in each muscle shows that the skewed motor unit size distribution holds for each sample.

Second, we found robust, quantitative relationships between axonal caliber, arbor length, and motor unit size. The cross-sectional area of an axon proximal to its intramuscular arborization scaled linearly with its intramuscular length (Figure 4D). Because axonal caliber is proportional to axoplasmic transport [52], it may scale with downstream metabolic expenditure. The energy expenditure is primarily devoted to resting and action potentials instead of synaptic transmission, therefore proportional to the surface area of the axonal membrane [53]. As long as the axonal caliber remains relatively constant, the surface area is proportional to arbor length. This may explain the linear relationship between axonal caliber and arbor length.

In addition, we found that the total intramuscular length of an axon scaled with the square root of its motor unit size (Figure 4E), akin to the prediction based on optimization considerations [54]. This power law scaling may be the result of the fact that average branch lengths progressively decreased as branch orders increased (Figure 4B). Therefore as motor unit size increases, the required increment in axonal arbor length is reduced. This relationship, combined with the proportionality between axonal caliber and arbor length, explains why axonal caliber scales sublinearly with motor unit size (Figure 4C).

Third, the axonal branching structure of each motor neuron was unique. We compared each axon with its functional counterparts, as defined by the size principle, in other muscles, and found substantial topological differences. Left-right pairs of corresponding neurons in the same animal showed no less variation than ipsi- or contralateral pairs from different animals. Such intra-animal variance is surprising, as each pair of neurons had identical genetic background and presumably experienced an identical environment. This result suggests that the branching pattern of these neurons was not predetermined, which contrasts strongly with the situation in invertebrates. For instance, the C. elegans connectome revealed remarkable stereotypy in the structure of the neural circuit. Worm neurons that are ontogenetic counterparts share almost identical branching patterns and connectivity both within an individual and across different animals, even though they may not be exact replicas of each other [18,19]. In annelids [55,56], insects [57–62], and crustaceans [63,64] individual neurons can also be identified, and their axonal branching patterns are stereotyped. In particular, this mammalian result contrasts with the stereotypy of neuromuscular innervation in invertebrates. For example, although there are fine structural differences in the terminal branching of axons at NMJs of any particular muscle fiber in insects, even these branches seem to have morphological regularities that are recognizable between different animals [65,66]. In mammals not only is the preterminal branching highly variable (as shown in this paper), but our experience suggests that no two NMJs look the same. Thus axonal branching in this mammalian system seems fundamentally different from that found in invertebrates.

Fourth, many axons exhibited tortuous trajectories en route to target muscle fibers, contrary to the notion that neural circuits should minimize total wiring length [67]. The layout of axonal arbors did help to minimize wiring length by preventing significant overlaps between territories of subtrees. However, other aspects of wiring, in particular extramuscular branches, wasted substantial wiring length. In contrast, in C. elegans neural wiring approximates the optimal solution fairly well [40]. The suboptimality in wiring length found in this work does not imply that the optimization principle per se is inapplicable; it rather suggests that factors other than wiring length also play a significant role. For instance, invertebrate nervous systems are under tight genetic control, and particular mutations in a single gene can lead to stereotyped alterations in neural wiring [62]. The mammalian neuromuscular system, on the other hand, may rely more strongly on activity-dependent reorganizations for each individual neural circuit to settle down on a particular wiring scheme. This strategy does not guarantee the establishment of optimal wiring, but only arrives at a solution that is functionally acceptable.

In conclusion, the interscutularis connectome reveals that in mammals, muscle function is implemented with a variety of wiring diagrams that share certain global features but differ substantially in anatomical form. Even the left and right copies of this neuromuscular circuit in the same animal exhibited significant variation. Nevertheless, the multitude of wiring diagrams exhibited no appreciable functional difference. Does this fact imply that, a posteriori, the observed variability in this system is inevitable, as there is no functional reason to impose a particular wiring diagram? We believe that this may not be the case. In general, the nervous system contains features that may have no adaptive value but tend to remain conserved [68,69]. This conservation of structure may be due to tight developmental constraints, as random changes during development may lead to dysfunction. Therefore, the rationale for the observed wiring variability may lie beyond the lack of functional significance.

This variability may result from the peculiarities of the nervous system of terrestrial vertebrates [13]. The neuromuscular circuit, for example, has a reduplicated arrangement of elements: each neuron belongs to a group of similar cells (the motor neuron pool) and projects to a population of similar postsynaptic targets (muscle fibers). At early developmental stages there is extensive fan-out (each neuron innervates a large number of muscle fibers) and fan-in (each muscle fiber is innervated by many neurons). The final circuit, however, retains only a small fraction of the initial connections—those that survived the pruning phase of synapse elimination [12,70]. This reorganization process is unidirectional (connections are lost but never regained), and the fate of an axonal branch is related to the identity of its competitors [27,28]. If a different input were eliminated from even one muscle fiber early on, there might be substantial divergence in the structure of the connectome when synapse elimination is complete. This sensitivity to developmental history may be the engine that generates diversity in neural wiring.

From this perspective, the variability is not a sign of lack of regulation, but rather indicates a different developmental strategy. Instead of genetically specifying the optimal wiring diagram for all individuals, this strategy allows a different instantiation to emerge in each case. Given the important role of interneuronal competition in the developing CNS [71–74], this strategy could well be a common theme in the entire mammalian nervous system. The value of this vertebrate innovation may be that it unfetters the structure of the nervous system from strict genetic determinism.

## Materials and Methods

### Sample preparation.

All animal experiments were performed according to protocols approved by Harvard University Institutional Animal Care and Use Committee (IACUC). Young adult (∼30 d old) transgenic mice of *thy-1*-YFP-16 line received IP injections of 0.1 ml/20g ketamine-xylazine (Ketaset, Fort Dodge Animal Health) or 0.2 ml/30 g sodium pentobarbital (64.8 mg/ml in sterile water). Once anesthetized, the animals were transcardially perfused with 4% paraformaldehyde (PFA) in 0.1 M phosphate-buffered saline (PBS [pH 7.4]). The interscutularis muscle along with a segment of its innervating nerve was removed and postfixed in 4% PFA for 30 min. Muscles were rinsed in PBS (25 °C, 30 min × 2) and mounted on slides with Vectashield mounting medium (Vector Laboratories). Mounted slides were slightly squeezed between a pair of small magnets for 12 h in order to flatten the tissue so that the distance from tissue surface to the coverslip was minimized and roughly constant.

### Muscle fiber-typing.

For identification of muscle fiber type, muscles were removed as above and postfixed with 1% PFA for 7 min, frozen, and sectioned at 20 μm using a Leica Cryostat. Then sections were incubated with blocking solution (2% BSA + 1% goat serum + 0.3% triton) at 25 °C for 3 h, and incubated with monoclonal antibodies against myosin type I and 2A (mouse anti-myosin I IgG1, 1:20, Novocastra; mouse anti-myosin 2A IgG1, 1:10, Iowa Hybridoma Bank) at 4 °C for 6–8 h. After several washes in 0.1% PBS-triton, samples were incubated with secondary antibody (Alexa-488 anti-mouse IgG1, 1:1,000; Molecular Probes) for 3 h. Finally muscle sections were rinsed in PBS (25 °C, 30 min × 2) and mounted on slides with Vectashield mounting medium.

### Confocal imaging.

Samples were imaged using a confocal laser scanning microscope (Zeiss Pascal, Carl Zeiss) equipped with a motorized stage. We used a 63× 1.4 NA oil-immersion objective and digitally zoomed-in so that each pixel was 0.1 μm (Nyquist limit). YFP florescence was excited with a 488-nm Argon laser and detected through a band-pass emission filter of 530–600 nm. The images were oversampled by a factor of 1.5 in the Z direction (Z-step sizes = 0.2 μm), with 12 bit dynamic range. Stack montages were obtained using the motorized stage controlled by the *MultiTimeZ* macro (Carl Zeiss), which set up the coordinates and imaging conditions for each stack. Adjacent stacks had 10% overlap to guarantee the precision of later alignment and tracing.

### Image processing and reconstruction.

Using custom-written Matlab (The MathWorks, Inc.) programs, image stacks were median-filtered and resized to 512 × 512 in XY to have cubic voxels (0.2 × 0.2 × 0.2 μm). Each stack was then digitally resampled along either *x*- or *y*-axis to generate a series of cross-sections that were approximately orthogonal to the direction of most axons. All axons were reconstructed from the series of cross-sections using custom-modified *Reconstruct* program (freely available from http://synapses.clm.utexas.edu/tools/reconstruct/reconstruct.stm; J. Lu, J. C. Fiala, J.W. Lichtman, unpublished data). Briefly, the modification incorporated a region-growing algorithm based on intensity threshold. The user presets the threshold and selects a point (seed) in an axon on one cross-section image. The program applies the region-growing algorithm to detect the contour of the axon on the image. Then it calculates the centroid of the contour, and propagates the centroid to the next image as the seed for the next cycle of edge-detection. The user can interrupt the progress of tracing at any time if aberrant region-growing occurs, and resets the threshold. Once all axons were traced out, they were rendered in 3D in *Reconstruct* and projected into 2D images (one image per axon), which were manually assembled into complete montages for each axon in Adobe Photoshop (Adobe Systems Inc.).

The 2D montage of each axon was retraced with the NeuronJ plug-in (http://www.imagescience.org/meijering/software/neuronj/ [75]) to ImageJ (http://rsb.info.nih.gov/ij/, NIH) to label and measure each axonal segment. The length and connectivity of axonal segments were transformed into a tree representation with custom-written Matlab programs.

### Verification of reconstruction accuracy.

Reconstruction accuracy was confirmed in three different ways. First, different persons independently traced a series of overlapping stacks, and the tracing results contained no gross-level discrepancy that would have led to different interpretations of the connectivity relationship between axonal branches. Second, we traced individual axons from tri-color mice using only one channel, and compared the results to the tri-color images. In the line of tri-color mouse (*thy-1*-KOFP × *thy-1*-YFP-H × *thy-1*-CFP-S), Kusabira-Orange fluorescent protein (KOFP) is expressed in 100% of motor axons (J. Livet and J.W. Lichtman, unpublished data); CFP and YFP are expressed each in a random subset of motor axons (24). Images were taken from all three fluorescent channels, and gray-level data from the KOFP channel only (Figure 1E) was used for reconstruction of axonal profiles. The monochromatic tracing results for doubly labeled axons (Figure 1F, yellow, KOFP + YFP; lavender, KOFP + CFP) were identical to that shown in the RGB images (Figure 1G, CFP, YFP, and KOFP were mapped to blue, green, and red, respectively). Third, we checked for abnormalities in tracing results such as axons looping back onto themselves or branches unconnected to any axon, and did not find any.

### Symmetry of axonal arbors.

We quantified the symmetry level of axonal arbors using the imbalance index *I*, which is defined as *I* = 2 × (∑_all interior nodes_ |*T*_R_ − *T*_L_|)/(*n* − 1)(*n* − 2). Here *T*_R_ and *T*_L_ are the number of terminals belonging to the right and left subtrees of the branching node, respectively, and *n* is the total number of terminals (NMJs) in the entire axon.

### Measurement of axonal caliber and arbor length.

Intramuscular axonal arbor length was defined as the total length downstream of a reference point common to all axons in the muscle. This reference point was chosen to be the first branching point of the axon that branched most proximally (close to cell body) in the muscle. Arbor length of axons that branched extramuscularly was defined to be the sum of arbor length of primary branches distal to the reference point. Axonal caliber was measured by dividing the volume *V* of an axonal segment (length ∼ 50 μm) slightly proximal to the reference point by the length of the segment. Volume was calculated as *V* = ∑_i_ *A*_i_ × *d*, where *A*_i_ was the area of the axonal profile on the *i*-th image section and *d* is section thickness. The calculated axonal caliber was normalized to the sum of calibers of all axons entering the muscle. Relationships between axonal caliber, arbor length, and motor unit size were fitted with GraphPad Prism 5 for Windows (GraphPad Software, Inc.).

### Quantifying spatial distribution of NMJs.

The spatial distribution of NMJs in each motor unit was parameterized along the lateral-medial axis (along muscle) and the rostral-caudal axis (across muscle). The relative position of a NMJ was defined as its rank order in the connectome along the axis. These relative positions were used to test whether a motor unit is distributed uniformly in the endplate band (Kolmogorov test [76]).

In order to test whether the two primary subtrees of an axon tend to “exclude” each other, we implemented the generalized Wald-Wolfowitz test in Matlab. For each axon, a minimal spanning tree (MST) was constructed from the distance between NMJs using Prim's algorithm [77]. Edges connecting NMJs of different subtrees were removed, and the number of resultant disjoint subgraphs was counted. Significance level *p* was obtained through a Monte Carlo simulation in which NMJs were reshuffled between subtrees. Two subtrees were considered completely segregated if removing one edge partitioned the MST into 2 disjoint subgraphs, each corresponding to a subtree.

### TED.

TED is defined as the minimal number of operations (insertion, deletion, and relabeling of nodes) required to transform one tree into another. TED was calculated with a custom implementation in Matlab of a dynamic programming algorithm. Tree structures of axons belonging to the right-side muscles were flipped horizontally so as to compare them with the ones on the left side, with which they would overlap if there were no branching differences.

We used Monte Carlo simulation to generate a large ensemble of axons all with the same number of terminals but random topologies consistent with the data. The simulation was based on a branching process model with level-dependent branching probabilities. In particular, probabilities of axons to terminate, bifurcate, trifurcate, etc, at each branching level were calculated from the full ensemble of reconstructed axons. 50 random “axons” were generated and their pair-wise TEDs (1,225 pairs in total) were compared to that of real axons with the same number of terminals.

## Supporting Information

Figure S1 An Additional Left/Right Pair of Interscutularis Connectomes This pair (M3L and M3R) had the same number of axons (16) and similar number of muscle fibers (left 195, right 196). Axons were color-coded according to the rank-order of their motor unit sizes in the respective connectome (A and B).
(9.76 MB PDF)

Figure S2 Higher Degree Branching in Axons (A) 3-furcation. In all panels, red arrow indicates the direction of the parent branch. Red arrowhead indicates the node of Ranvier where the branching occurs. Each child branch is marked with a red numeral.
(B) 4-furcation. Two of the child branches crossed each other.
(C) 5-furcation. Notice that one of the child branches sat above another child branch (blue arrow) so the two appear merged in the XY projection.
(4.81 MB PDF)

Figure S3 Length Distribution of Axonal Segments for Different Branch Orders Axonal segments of every branch order exhibited a wide range of lengths, with a predominance of shorter segments over longer ones. Segments of different branch orders exhibited different length distributions (*p* < 0.0001, Kruskal-Wallis test). Boxes, 25%, 50%, and 75% quartiles; horizontal bars, minimum and maximum lengths. Number of segments in the group indicated above the top bar.
(243 KB PDF)

Figure S4 Location of Innervation Sites of Each Axon in an Interscutularis Muscle Locations of NMJs in each motor unit were parameterized along the lateral-medial axis (along muscle) and the rostral-caudal axis (across muscle). Relative positions of NMJs along each axis were shown (ladders) against the entire connectome (gray stripe). Ladders were color-coded according to motor unit size as in Figure 3.
(4.14 MB PDF)

Figure S5 An Axon Whose Primary Subtrees Invaded Disjoint Territories NMJs innervated by one subtree (in orange) are marked by red dots; NMJs innervated by the other subtree (in cyan) are marked by green dots. Lines connecting dots are the minimal-spanning tree (MST) constructed from the coordinates of NMJs (see Methods for details). If the edge marked as the yellow dotted line is removed, the MST is partitioned into two disjoint subtrees, each corresponding to one subtree of the original axonal arbor.
(333 KB PDF)

Figure S6 The Number of Both Muscle Fibers and Innervating Axons in Left-Right Muscle Pairs Exhibited No Systematic Difference (A) The degree of disparity in the number of muscle fibers in six left/right pairs of interscutularis muscles (M3–M8). Horizontal bars indicate the average value of each pair. No systematic difference between left and right muscles was detected (two-tailed *p* = 0.53, paired Student's *t*-test).
(B) The number of motor axons innervating the left and right muscles was not significantly different (two-tailed *p* = 0.47, paired Student's *t*-test).
(265 KB PDF)

## Abbreviations

CI: confidence interval
CNS: central nervous system
NMJ: neuromuscular junction
PNS: peripheral nervous system
TED: tree-editing distance
