# Correction: The Interscutularis Muscle Connectome

**DOI:** 10.1371/journal.pbio.1000108

**Published:** 2009-04-28

**Authors:** Ju Lu, Juan Carlos Tapia, Olivia L White, Jeff W Lichtman

Correction for:

Citation: Lu J, Tapia JC, White OL, Lichtman JW (2009) The interscutularis muscle connectome. PLoS Biol 7(2): e1000032. doi:10.1371/journal.pbio.1000032

In the Materials and Methods section titled “Confocal imaging,” there was an error in the second sentence. The correct sentence should read: “We used a 63× 1.4 NA oil-immersion objective and optically zoomed-in so that each pixel was 0.1 μm (Nyquist limit).”

