# Supplementary material for: The Interscutularis Muscle Connectome
Source: PLoS Biol. 2009 Feb 10;7(2):e1000032. doi: 10.1371/journal.pbio.1000032 (PMC2637925; doi:10.1371/journal.pbio.1000032)
Supplement: Supplementary file 1 [file pbio.1000032.sg001.pdf]

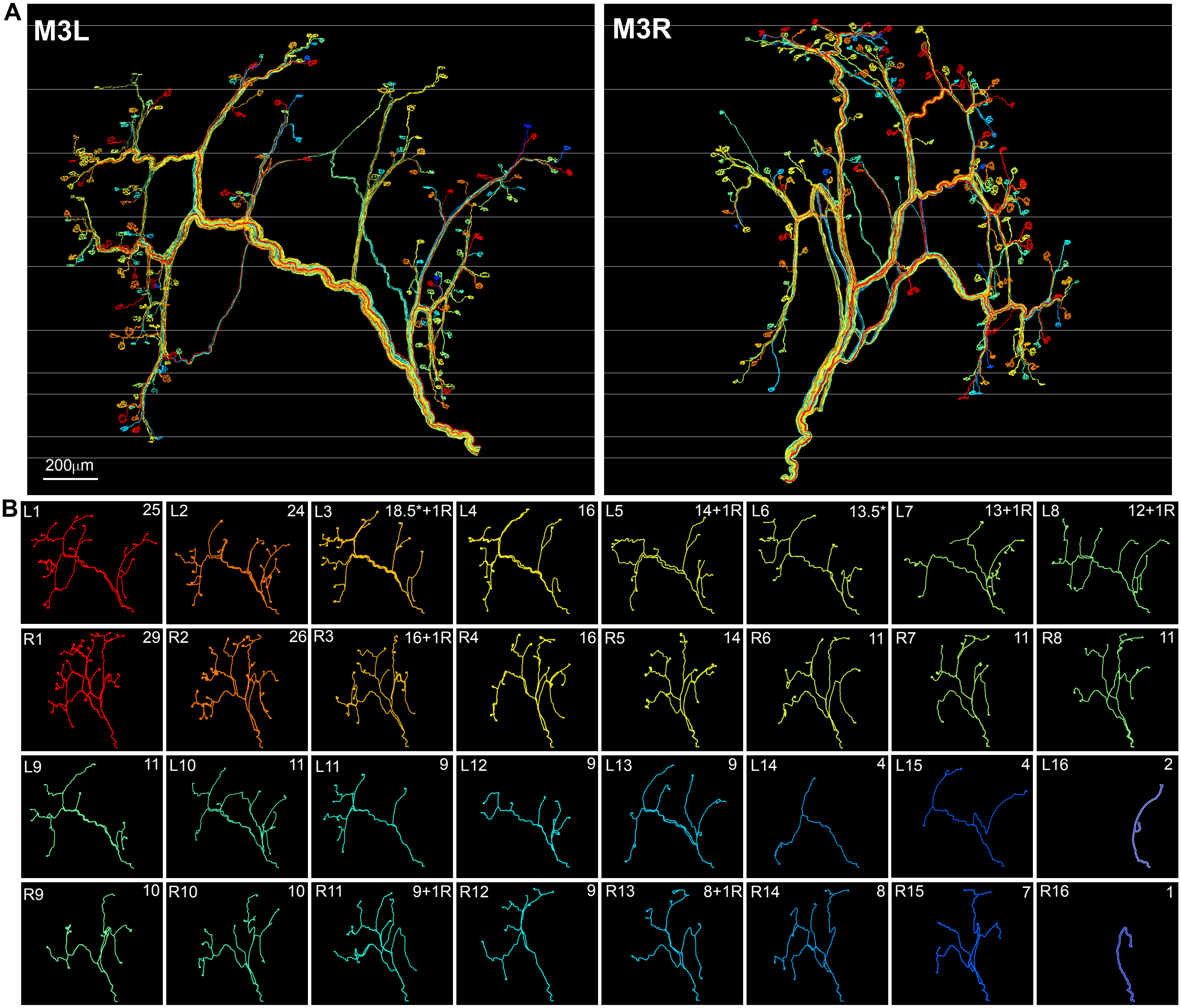
A
M3L
M3R
200μm
B
L1 25
L2 24
L3 18.5*+1R
L4 16
L5 14+1R
L6 13.5*
L7 13+1R
L8 12+1R
R1 29
R2 26
R3 16+1R
R4 16
R5 14
R6 11
R7 11
R8 11
L9 11
L10 11
L11 9
L12 9
L13 9
L14 4
L15 4
L16 2
R9 10
R10 10
R11 9+1R
R12 9
R13 8+1R
R14 8
R15 7
R16 1
