# Supplementary material for: The Interscutularis Muscle Connectome
Source: PLoS Biol. 2009 Feb 10;7(2):e1000032. doi: 10.1371/journal.pbio.1000032 (PMC2637925; doi:10.1371/journal.pbio.1000032)
Supplement: Supplementary file 2 [file pbio.1000032.sg002.pdf]

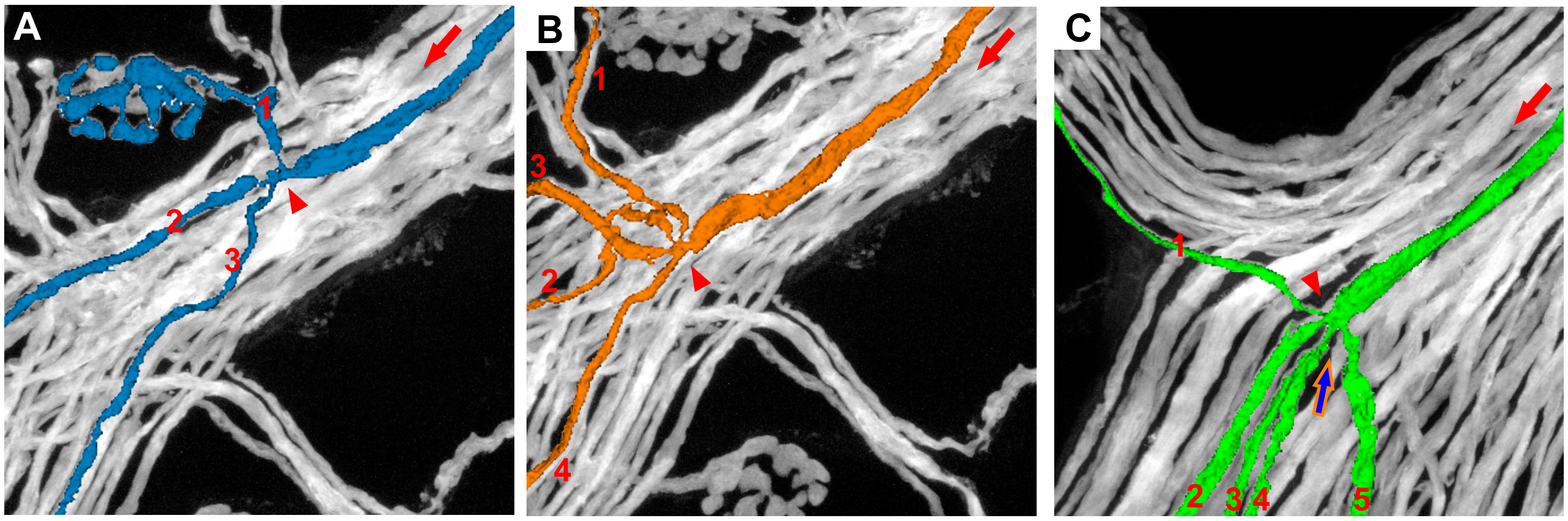
A
1
2
3
B
1
3
2
4
C
1
2
3
4
5
