# Supplementary material for: The Interscutularis Muscle Connectome
Source: PLoS Biol. 2009 Feb 10;7(2):e1000032. doi: 10.1371/journal.pbio.1000032 (PMC2637925; doi:10.1371/journal.pbio.1000032)
Supplement: Supplementary file 3 [file pbio.1000032.sg003.pdf]

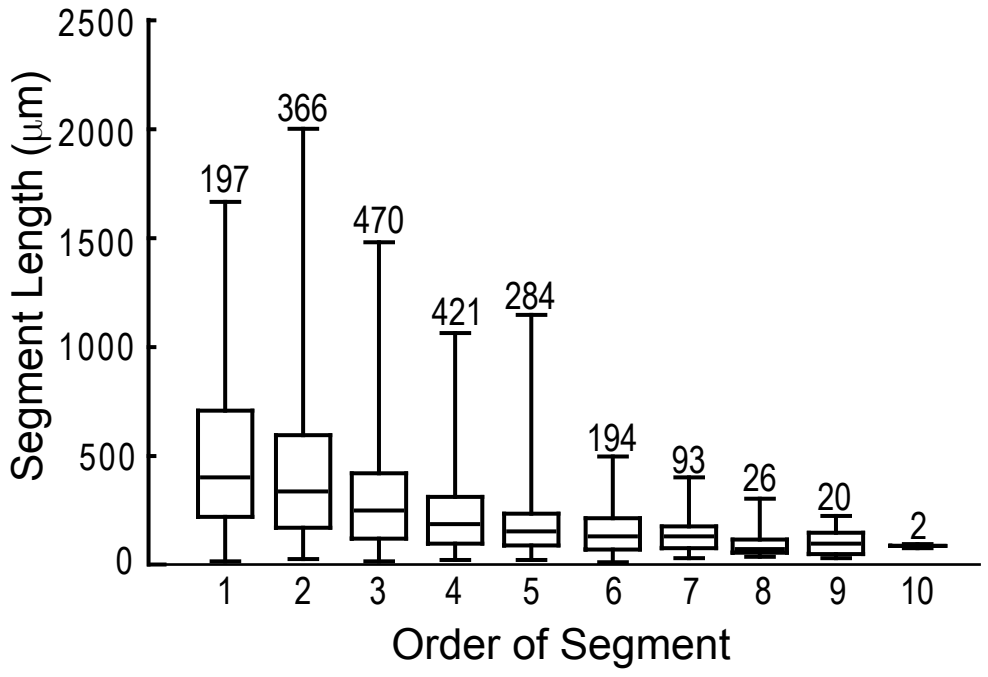

Segment Length (μm)
2500
2000
1500
1000
500
0
197
366
470
421
284
194
93
26
20
2
1
2
3
4
5
6
7
8
9
10
Order of Segment
