# Supplementary material for: The Interscutularis Muscle Connectome
Source: PLoS Biol. 2009 Feb 10;7(2):e1000032. doi: 10.1371/journal.pbio.1000032 (PMC2637925; doi:10.1371/journal.pbio.1000032)

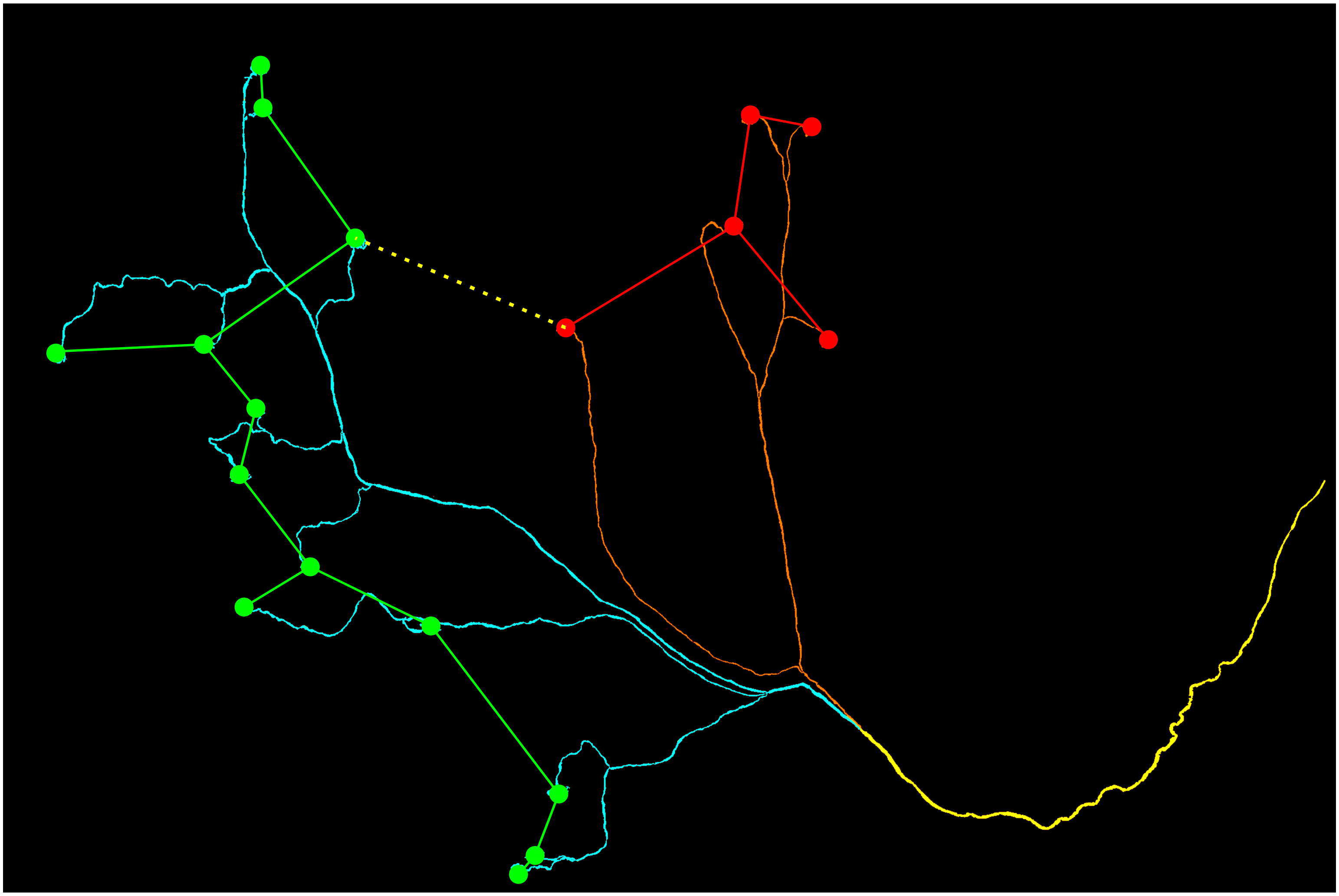

Supplement: Supplementary file 5 [file pbio.1000032.sg005.pdf]
