# Supplementary material for: The Interscutularis Muscle Connectome
Source: PLoS Biol. 2009 Feb 10;7(2):e1000032. doi: 10.1371/journal.pbio.1000032 (PMC2637925; doi:10.1371/journal.pbio.1000032)
Supplement: Supplementary file 6 [file pbio.1000032.sg006.pdf]

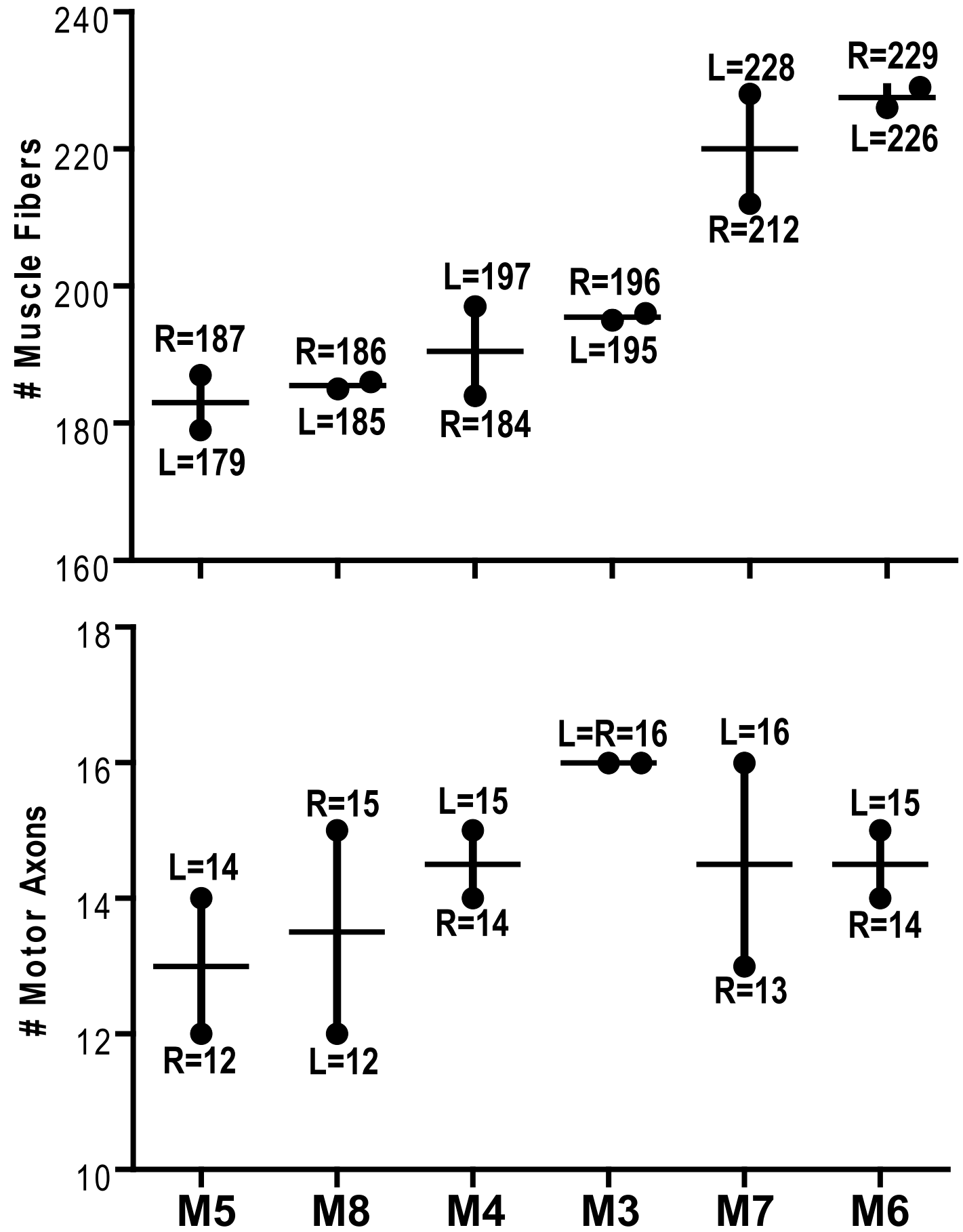

Muscle Fibers
240
220
200
180
160
R=187
L=179
R=186
L=185
L=197
R=184
R=196
L=195
L=228
R=212
R=229
L=226
# Motor Axons
18
16
14
12
10
L=14
R=12
R=15
L=12
L=15
R=14
L=R=16
L=16
R=13
L=15
R=14
M5
M8
M4
M3
M7
M6
